# Glucocorticoids in the treatment of non-infectious superior ophthalmic vein thrombosis – Three cases and a review of the literature

**DOI:** 10.1016/j.ajoc.2024.102027

**Published:** 2024-03-02

**Authors:** Samuel Sigurdsson, Elin Bohman, Frank Träisk, Urszula Arnljots

**Affiliations:** a St. Erik Eye Hospital, Stockholm, Sweden; b Division of Eye and Vision, Department of Clinical Neuroscience, Karolinska Institutet, Stockholm, Sweden

**Keywords:** Superior ophthalmic vein thrombosis, SOVT, Cavernous sinus thrombosis, CST, Treatment, Glucocorticoids

## Abstract

**Purpose:**

Superior ophthalmic vein thrombosis (SOVT) is a rare clinical entity, which can have a septic and an aseptic cause. Aseptic SOVT is typically treated with anticoagulation. Glucocorticoids are reserved for cases with concurrent orbital inflammation.

We present three cases of SOVT due to carotid cavernous fistula not responding to standard treatment, subsequently successfully treated with glucocorticoids.

**Observations:**

Three patients with various degrees of proptosis, ophthalmoplegia, orbital stasis and reduced vision are presented. One patient was confirmed to have isolated SOVT, while the other two had associated cavernous sinus thrombosis. All patients had underlying carotid-cavernous fistula without signs of infection. All patients were initially treated with parenteral anticoagulation. Two patients were treated with intraocular pressure-reducing medication. One of whom underwent canthotomy-cantholysis. Two patients experienced a gradual worsening of symptoms during treatment with anticoagulation, while one patient improved before deteriorating. All patients received additional treatment with glucocorticoids consisting of a three-day treatment with intravenous methylprednisolone 500 mg, followed by oral glucocorticoids resulting in total regression of symptoms. Two patients regained 20/20 vision, with some vision field defects, while the third patient regained 20/25 vision.

**Conclusion and importance:**

The addition of glucocorticoids in the treatment of aseptic SOVT can lead to improvement of symptoms and a potentially better prognosis. However, the risk of complications of glucocorticoid treatment must be carefully considered on a case-by-case basis.

## 1 Introduction

Superior ophthalmic vein thrombosis (SOVT) is a rare yet potentially fatal, condition with an estimated yearly incidence of approximately three to four cases per one million.^1^ The underlying cause can be both septic and aseptic, the latter being more common.^1^, ^2^, ^3^

Treatment for SOVT is challenging and varies based on its etiology. Anticoagulant therapy is standard therapy for aseptic SOVT as well as in cases with progression to cavernous sinus thrombosis (CST).^2^ In cases with associated orbital inflammation, treatment with glucocorticoids with or without anticoagulant treatment has been used successfully.^2^ However, glucocorticoids have not been routinely used in the treatment of non-orbital inflammation associated (non-OI) SOVT.^3^

This case series describes three patients with aseptic non-OI SOVT associated with underlying carotid-cavernous fistulas (CCF) not responding to standard therapy showing improvement after intravenous glucocorticoids.

Informed written consent was obtained and the study was performed in accordance with the Helsinki Declaration.

## 2 Case description

### 2.1 Case 1

**Fig. 1:**
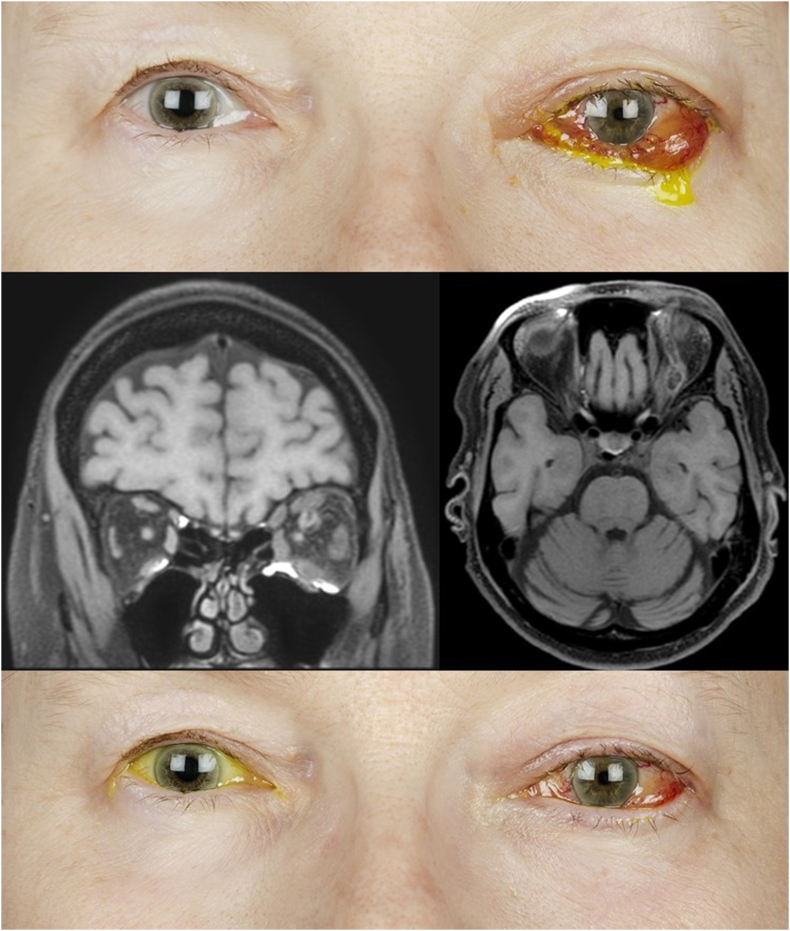
Case 1 – Patient with superior ophthalmic vein thrombosis and cavernous sinus thrombosis. **A** Photo at initial presentation. **B–C** Magnetic resonance imaging at initial presentation showing enlarged superior ophthalmic vein, orbital congestion and enlarged ocular muscles. **D** Photo at follow up three weeks later.

A 64-year-old previously healthy woman presented with a few months’ history of ocular irritation, epiphora and left eyelid swelling. A few days preceding the first visit she experienced acute worsening with pain, diplopia, proptosis, and decreased vision (Fig. 1 A). Visual acuity (VA) in the left eye (OS) was counting fingers, and intraocular pressure (IOP) was 60 mm Hg. A left relative afferent pupillary defect (RAPD) was present. Anterior segment examination showed eyelid edema, total ptosis, diffuse hemorrhagic chemosis and corneal edema. Initial posterior segment examination was difficult due to corneal status. Exophthalmometry showed 10 mm proptosis OS with total ophthalmoplegia. Visual acuity and examination of the right eye (OD) was normal. The patient had no fever and blood tests gave no reason to suspect an infectious cause. Magnetic resonance imaging (MRI) of the orbit (Fig. 1B–C) revealed a SOVT, CST and edematous structures within the left orbit. A carotid-cavernous fistula was suspected. Urgent canthotomy with cantholysis was performed, with IOP reduction to 27 mmHg. Treatment with anticoagulation, dalteparin (100 IU/kg a day) was initiated in addition to eye-pressure reducing medication. On follow-up the next day clinical status had marginally improved, with decreased eyelid edema, conjunctival chemosis and corneal edema. Dilated retinal veins were noted on fundus exam. Conventional angiography confirmed an indirect fistula between the left internal carotid artery and cavernous sinus. An attempt to embolize the fistula was performed, but failed. On the sixth day of hospitalization, VA was still counting fingers and IOP was 30 mmHg, while eye motility had slightly improved and proptosis had reduced to 7 mm. Due to the limited efficacy of conventional treatment during the first six days, intravenous (IV) methylprednisolone (500 mg daily for three days) was initiated. This was followed by oral prednisolone (initial dose of 40 mg daily with slow tapering). On the third day of the methylprednisolone treatment, clinical improvement was observed. Visual acuity was 5/20 in the left eye, IOP was 27 mmHg and three mm proptosis remained. No RAPD was observed, and the eye was less swollen. On the three-month follow-up, VA in the left eye was 20/20, IOP was 12 mmHg, ocular motility was normal with complete resolution of proptosis (Fig. 1 D). The visual field (Humphrey® Field Analyzer 3, Carl Zeiss Meditec Inc, Dublin, CA, USA) showed minimal diffuse depressions with visual field index (VFI) 97%.

### 2.2 Case 2

**Fig. 2:**
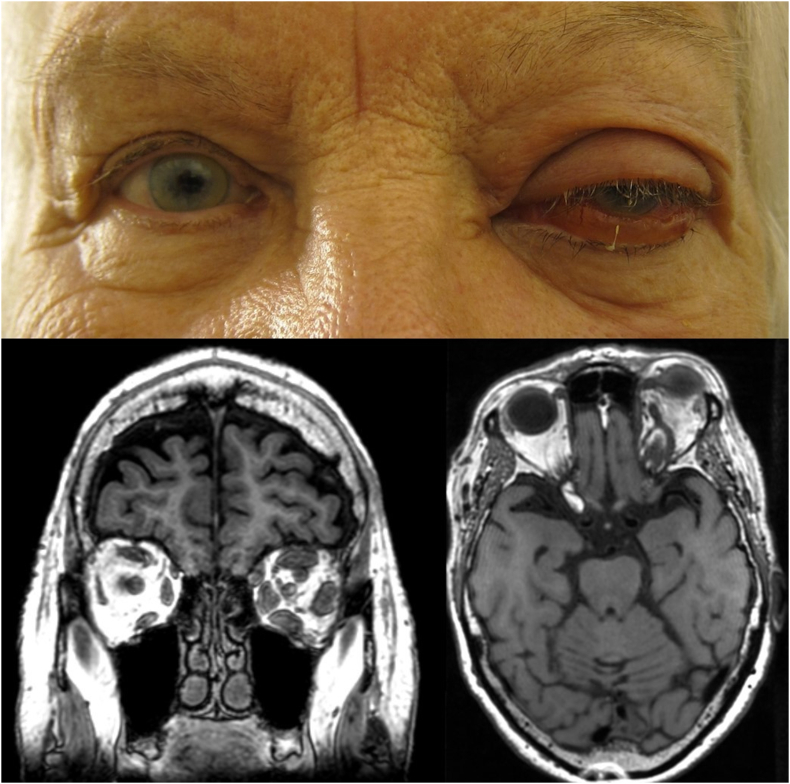
Case 2 – Patient with isolated superior ophthalmic vein thrombosis. **A** Photo at initial presentation. **B–C** Magnetic resonance imaging at initial presentation showing enlarged superior ophthalmic vein, orbital congestion and enlarged ocular muscles.

An 84-year-old woman with a previously diagnosed CSF, presented with a three-day history of a sudden onset left sided ptosis, proptosis, swelling and redness of the eye (Fig. 2 A). Visual acuity and IOP were 20/20 and 10 mm Hg OD and 20/25 and 25 mm Hg OS, respectively. There was no RAPD, but color vision was affected in OS. There were impairments of abduction and depression as well as 11 mm proptosis in OS. The left eyelid was swollen, with ptosis reaching the pupillary plane. Conjunctival injection and chemosis were moderate and the optic nerve head was hyperemic without edema. Vital signs were normal and blood tests elicited no suspicion of infection. Investigations with MRI and conventional angiography showed a direct carotid-cavernous fistula and SOVT. The patient was initially treated with intraocular pressure-lowering eye-drops and IV heparin for the first three days, followed by oral apixaban (2,5 mg x 2). Improvement was noted within the next few days, with a reduction of proptosis to six mm on discharge on the eighth day.

On follow-up four weeks later the patient complained of worsening of vision and aggravation of symptoms during the preceding two weeks, despite continuing apixaban. Visual acuity and IOP OS were 20/200 and 13 mm Hg, respectively. Clinical findings OS included 10 mm proptosis, subtotal ophthalmoplegia, presence of RAPD, increased periorbital edema, profuse chemotic hemorrhagic conjunctiva, optic nerve swelling with flame hemorrhages and retinal edema. Magnetic resonance imaging (Fig. 2B–C) showed signs of SOVT with increased intraorbital edema. The patient was treated with IV methylprednisolone (500 mg daily for three days), followed by betamethasone (six mg with one-week tapering) in addition to apixaban. Two days later vision started to improve, and the periorbital edema was reduced to the level where the patient could spontaneously open the left eye again. Further improvements in vision and symptoms were noted during the next few days. Apixaban was switched to subcutaneous dalteparin at discharge. Six weeks later VA was 20/50, with a remaining six mm proptosis, discrete abduction deficit in OS.

Six months later VA and IOP OS were 20/25 and nine mm Hg, respectively, with two mm proptosis remaining and no RAPD or motility deficits. Examination revealed dilated episcleral blood vessels and a few retinal dot hemorrhages but was otherwise unremarkable.

### 2.3 Case 3

**Fig. 3:**
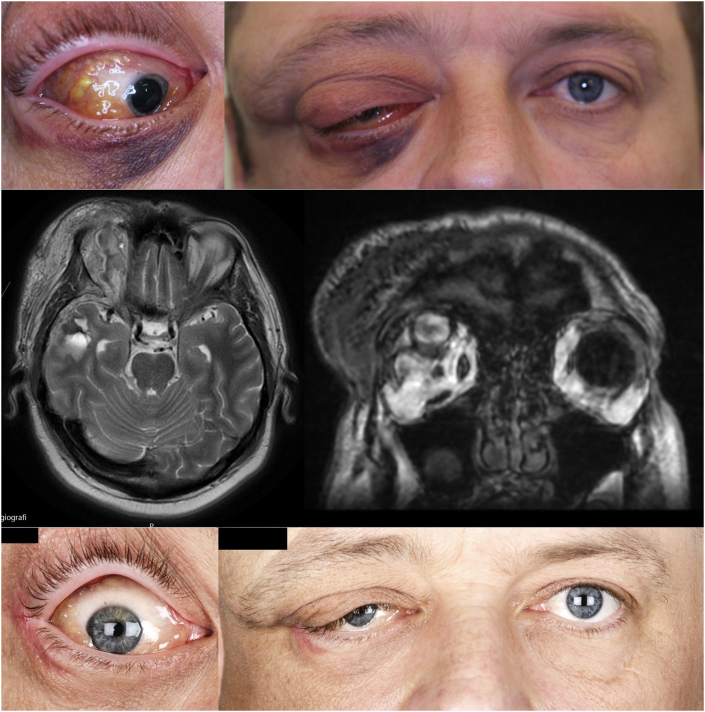
Case 3 – Patient with superior ophthalmic vein thrombosis and cavernous sinus thrombosis. **A-B** Photos at initial presentation. **C-D** Magnetic resonance imaging at initial presentation showing enlarged superior ophthalmic vein, orbital congestion and enlarged ocular muscles **E-F** Follow-up 10 weeks later.

A 44-year-old man with type 2 diabetes, lymphatic-venous malformation in the right orbit and face and proptosis, presented with a seven-day history of gradual worsening of headache, right sided eye pain and periorbital swelling (Fig. 3A–B). Visual acuity and IOP were 20/20 and 20 mm Hg in both eyes, respectively. No RAPD nor impairment of color vision were noted. Eye movements were mildly impaired and OD proptosis measured 4.5 mm. The right eyelid was S-shaped, with a total ptosis temporally, but just over the pupil-plane centrally (Fig. 3-B). The conjunctival injection and chemosis were moderate and fundoscopy unremarkable. The patient had normal vital signs and blood tests elicited no suspicion of infection.

Magnetic resonance imaging (Fig. 3C–D) and conventional angiography confirmed a SOVT, CST and a shunt from the right medial meningeal artery to the venous system. Symptoms progressed despite initial treatment with dalteparin (200 IU/kg),. On the fourth day of admission, VA had decreased to 20/30 in OD. There was a significant impairment of color vision and a RAPD OD. Intraocular pressure was normal. Ocular motility showed impairment of elevation and adduction, along with partial impairment in abduction. Proptosis OD was six mm. There was profuse eyelid swelling and conjunctival chemosis. Intravenous methylprednisolone (500 mg daily for three days) was initiated, followed by prednisolone (20 mg daily for three weeks). Gradual improvement was noted in symptoms and signs over the next few days.

Seven months after admission, proptosis had returned to its previous state (Fig. 3E–F). Ocular motility was normal. Visual acuity was 20/20 OU, although visual field examination showed a nasal defect OD (VFI 78%). The optic nerve head was pale and macular optical coherence tomography showed thinning of the ganglion cell layer.

## 3 Discussion

We report three cases of non-infectious SOVT associated with carotid-cavernous fistula, with periorbital swelling, ophthalmoplegia and optic neuropathy. One of the patients had initial improvement of symptoms but deteriorated during continuous anticoagulation therapy, only exhibiting marked improvement after initiation of glucocorticoids. The other two had limited effect of treatment until the addition of glucocorticoid treatments. Two of the patients regained VA 20/20 although with some remaining visual field defects and one patient regained VA 20/25.

In the International Study on Cerebral Veins and Dural Sinus Thrombosis (ISCVT), a large multinational study of 624 adult patients with cerebral vein thrombosis, glucocorticoid treatment was shown not to be beneficial in terms of morbidity and mortality, with a trend towards worse outcomes in patients without a parenchymal lesion. The study did, however, only include eight patients with thrombi in the cavernous sinus.^4^ In cases of cavernous sinus thrombosis, evidence is more unclear.^5^ Treatment with corticosteroids has not been shown to have an effect on morbidity and mortality in CST, although possibly beneficial in reducing orbital congestion and improving cranial nerve function.^6^ The use of glucocorticoids may reduce orbital edema in some patients, consequently relieving pressure on orbital structures and reducing structural damage during dissolvement of the thrombi.^7^^,^^8^ Furthermore, reduction of external pressure on the superior ophthalmic vein might improve venous flow, leading to further reduction of edema.

However, the prothrombotic effects of glucocorticoids must be carefully considered, as there is more than a three-fold increase in the risk of venous thrombus formation in patients treated with glucocorticoids.^9^

For isolated SOVT or even SOVT with concurrent CST there is a paucity of high-quality data on glucocorticoid treatment, although well established in SOVT associated with orbital inflammation.^2^^,^^3^Multiple case reports have previously reported the use of glucocorticoid treatment in aseptic, non-OI SOVT. Two case reports described patients with SOVT and a previous history of deep vein thrombosis who recently terminated their anticoagulation therapy.^10^^,^^11^ In both cases the patients presented with no light perception VA, treated with IV glucocorticoids and anticoagulation without visual improvement, although proptosis and ophthalmoplegia resolved. In another report, a patient with a previous history of autoimmune hemolytic anemia presented with proptosis, periorbital ecchymosis, conjunctival and episcleral congestion and subconjunctival hemorrhage due to isolated SOVT.^12^ This patient had normal vision, pupillary reaction and eye movements. After treatment with IV methylprednisolone, the patient's symptoms gradually resolved. Another patient is reported to have developed bilateral isolated SOVT following a high altitude mountain climbing.^13^ After treatment with oral glucocorticoids and anticoagulation symptoms regressed. Two patients with previously undiagnosed systemic lupus erythematosus presented acutely with reduced vision, proptosis and ophthalmoplegia due to SOVT.^14^^,^^15^ Both patients were treated with glucocorticoids and anticoagulation, which resulted in marked improvement. Finally, seven patients with SOVT following carotid compression or stent-assisted coil embolization received glucocorticoids in addition to conventional therapy.^16^^,^^17^ Four of these patients recovered to a VA of 20/20, while one of the patients, who suffered compressive optic neuropathy before initiation of therapy, recovered to a VA of 20/60. Of the two patients with post coiling SOVT, one doubled his VA to 20/200 while the other retained his presenting VA of 20/200. Both patients had resolution of other symptoms.

In all reported cases treatment with glucocorticoid was successful and ten of the fifteen patients regained good vision. There was no reported worsening of ocular symptoms or other major side effects of the steroid treatment. This suggests that the addition of glucocorticoids may be beneficial in refractive cases of aseptic non-OI SOVT of several different etiologies, including SOVT caused by arterio-venous fistulas. However, as stated above, treatment is not without the risk of complications. Therefore, careful consideration on a case-by-case basis is crucial.

Although one could argue that our patients might have regained the function even without the glucocorticoids, the prompt and marked improvement after initiating the treatment suggests meaningful beneficial additive effect.

Until a large multi-center prospective study is conducted, clinical cases such as these are an important addition to the literature, helping guide providers with decision-making in difficult rare cases.

## Ethics approval and consent to participate

Written informed consent was obtained from all patients included in this study. The study was approved by the Swedish Ethical Review Authority (dnr 2023-01548-01).

## Consent for publication

All authors consent to the publication of this manuscript to American Journal of Ophthalmology Case Reports.

## Availability of data and materials

Not applicable.

## Authorship

All authors meet the ICMJE criteria for authorship.

## CRediT authorship contribution statement

**Samuel Sigurdsson:** Conceptualization, Writing – original draft, Writing – review & editing. **Elin Bohman:** Conceptualization, Writing – review & editing. **Frank Träisk:** Conceptualization, Writing – review & editing. **Urszula Arnljots:** Conceptualization, Writing – review & editing.

## Declaration of competing interest

The authors declare that they have no known competing financial interests or personal relationships that could have appeared to influence the work reported in this paper.

## References

[1] Sotoudeh H., Shafaat O., Aboueldahab N. (2019). Superior ophthalmic vein thrombosis: what radiologist and clinician must know?. Eur J Radiol Open.

[2] Adam C.R., Shields C.L., Gutman J. (2018). Dilated superior ophthalmic vein: clinical and Radiographic features of 113 cases. Ophthalmic Plast Reconstr Surg.

[3] van der Poel N.A., de Witt K.D., van den Berg R. (2019). Impact of superior ophthalmic vein thrombosis: a case series and literature review. Orbit.

[4] Canhão P., Cortesão A., Cabral M. (2008). Are steroids useful to treat cerebral venous thrombosis?. Stroke.

[5] Desa V., Green R. (2012). Cavernous sinus thrombosis: current therapy. J Oral Maxillofac Surg.

[6] Weerasinghe D., Lueck C.J. (2016). Septic cavernous sinus thrombosis: case report and review of the literature. Neuro Ophthalmol.

[7] Friberg T.R., Sogg R.L. (1978). Ischemic optic neuropathy in cavernous sinus thrombosis. Arch Ophthalmol.

[8] Ebright J.R., Pace M.T., Niazi A.F. (2001). Septic thrombosis of the cavernous sinuses. Arch Intern Med.

[9] Orsi F.A., Lijfering W.M., Geersing G.J. (2021). Glucocorticoid use and risk of first and recurrent venous thromboembolism: self‐controlled case‐series and cohort study. Br J Haematol.

[10] Lim L.H., Scawn R.L., Whipple K.M. (2014). Spontaneous superior ophthalmic vein thrombosis: a rare entity with potentially devastating consequences. Eye.

[11] Mandić J.J., Mandić K., Mrazovac D. (2018). Superior ophthalmic vein thrombosis with complete loss of vision as a complication of autoimmune and infective conditions. Ocul Immunol Inflamm.

[12] Rao R., Ali Y., Nagesh C. (2018). Unilateral isolated superior ophthalmic vein thrombosis. Indian J Ophthalmol.

[13] Shahlaee A., Hennein L.M., Winn B.J. (2021). Bilateral superior ophthalmic vein thrombosis associated with high altitude. Orbit.

[14] Sambhav K., Shakir O., Chalam K.V. (2015). Bilateral isolated concurrent superior ophthalmic vein thrombosis in systemic lupus erythematosus. Int Med Case Rep J.

[15] Dey M., Charles Bates A., McMillan P. (2013). Superior ophthalmic vein thrombosis as an initial manifestation of antiphospholipid syndrome. Orbit.

[16] Salim S., Koka K., Halbe S. (2021). Superior ophthalmic vein thrombosis post manual carotid compression for indirect carotid-cavernous fistula. Orbit.

[17] Abri Aghdam K., Nilforushan N., Zand A. (2020). Superior ophthalmic vein thrombosis following coil embolization of posterior communicating artery aneurysm. Report of two cases.

